# Ellis-van Creveld Syndrome: A Case Report

**DOI:** 10.5005/jp-journals-10005-1138

**Published:** 2012-02-24

**Authors:** Subash Singh, Vandana Arya, M Jonathan Daniel, Vijeev Vasudevan

**Affiliations:** Lecturer, Department of Pedodontics and Preventive Dentistry Mahatma Gandhi Post Graduate Institute, Puducherry-605006, India e-mail: vasu11pedodontics@rediffmail.com; Lecturer, Department of Oral Medicine and Radiology, Maharana Pratap College of Dentistry and Research Centre, Gwalior, Madhya Pradesh, India; Professor and Head, Department of Oral Medicine and Radiology Mahatma Gandhi Post Graduate Institute, Puducherry, India; Professor, Department of Oral Medicine and Radiology Krishnadevaraya College of Dental Sciences and Hospital, Bengaluru Karnataka, India

**Keywords:** Chondroectodermal dysplasia, Polydactyly, Congenital heart disease

## Abstract

Ellis-van Creveld syndrome also known as chondroectodermal dysplasia or mesoectodermal dysplasia; a rare genetic disorder of the skeletal dysplasia. ‘Six-fingered dwarfism’ (digital integer deficiency) was an alternative designation used for this condition when it was being studied in the Amish. It is characterized by short-limb dwarfism, polydactyly, malformation of the bones of the wrist, dystrophy of the fingernails, partial hare-lip, cardiac malformation and often prenatal eruption of the teeth. A typical case of Ellis-van Creveld syndrome is reported in the present article.

**How to cite this article:** Singh S, Arya V, Daniel MJ, Vasudevan V. Ellis-van Creveld Syndrome: A Case Report . Int J Clin Pediatr Dent 2012;5(1):72-74.

## INTRODUCTION

Ellis-van Creveld syndrome (EVCS) is also known as chondroectodermal dysplasia or mesoectodermal dysplasia, is a rare autosomal recessive disorder, caused by mutations in the EVC and EVC2 gene (4p16), mapped to the short arm of chromosome 4.^2^ The disorder was described by Richard WB Ellis (1902–1966) of Edinburgh and Simon van Creveld (1895–1971) of Amsterdam.^11^ A large number of cases were reported in the Amish community of Lancaster, Pennsylvania, USA by McKusick in 1964.^4^Today this syndrome has been described in other populations and it is known to affect all races. EVCS presents with the characteristic tetrad of: (1) Disproportionate dwarfism with short limbs and exceptionally long trunk, (2) bilateral postaxial polydactyly of the hands, (3) dystrophic nails, hypodontia and malformed teeth, (4) congenital cardiac malformations occur in 50 to 60% of cases, most common being the interseptal defect.^1-3^Incidence in India is very rare. Only single case report has been found (Popli and Popli 2002).^10^ This report describes a case of EVC syndrome in a 10-year-old boy with the tetrad of principal features.

## CASE REPORT

A 10-year-old male patient reported to the Department of Pedodontics and Preventive Dentistry, MGPGI, Puducherry, with the chief complaint of missing teeth. Patient was the second child of healthy parents of consanguinous marriage.

**Fig. 1:**
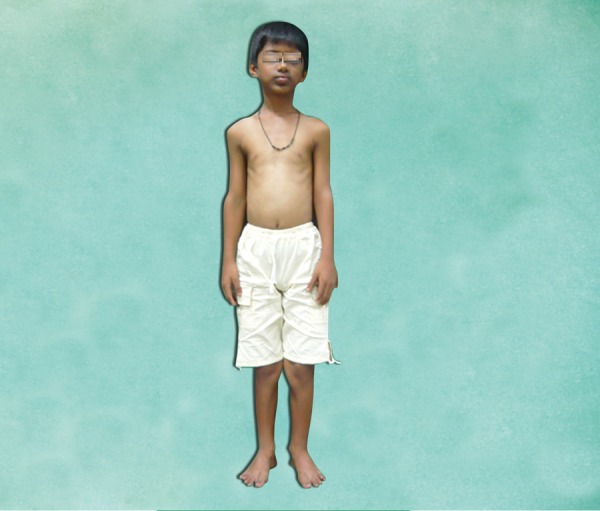
Short stature and genu valgum

**Fig. 2:**
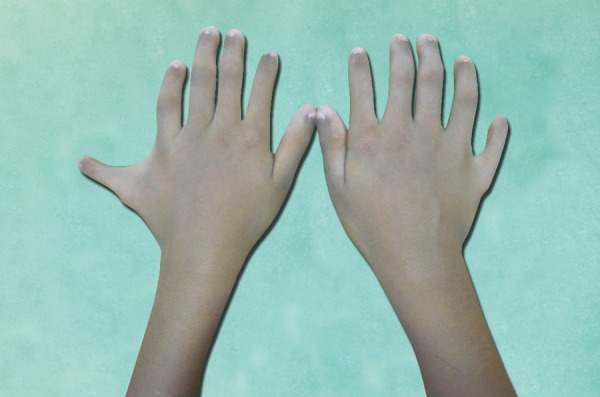
Hands showing six digits and small, brittle nails

Dental history revealed unerupted maxillary and mandibular front teeth ever and there was no history of extraction and spontaneous exfoliation of any teeth. Medical history revealed early exertion after routine work for past 6 months. No other significant systemic illness was found. He had normal IQ level. General examination revealed that the patient was of short stature (height 126 cm), thin build (21 kg) and shortening of upper arm with the arm span of 120 cm (Fig. 1). The examination of hand revealed hypoplastic nails and polydactyly in both the hands (Fig. 2). Arms and legs were short and thickened. Extraoral examination revealed normal head morphology and hair appeared normal in quantity and quality. There was no obvious asymmetry in face. Maxilla was mildly hypoplastic.

**Fig. 3:**
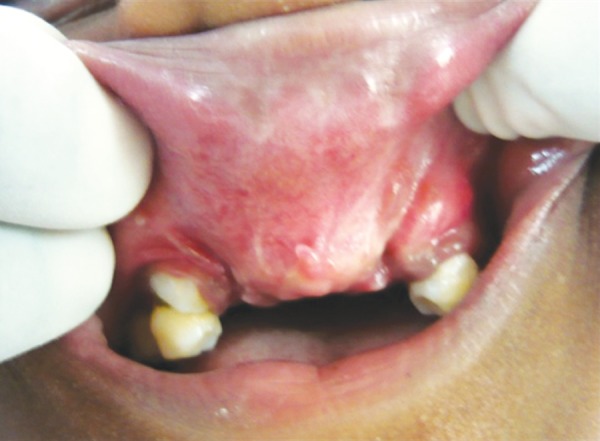
Labiogingival attachment in maxillary arch

**Fig. 4:**
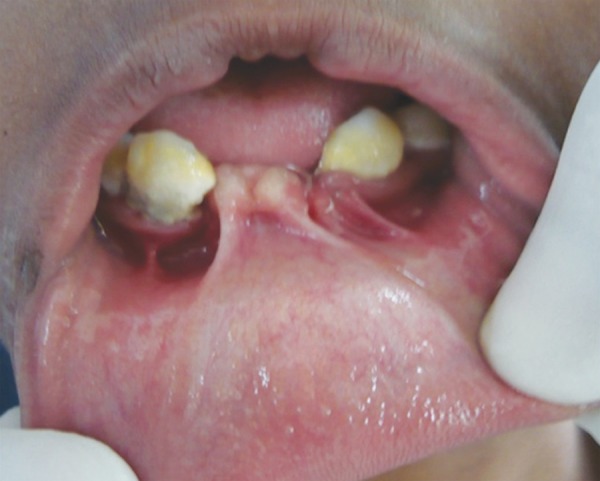
Labiogingival attachment in mandibular arch

**Fig. 5:**
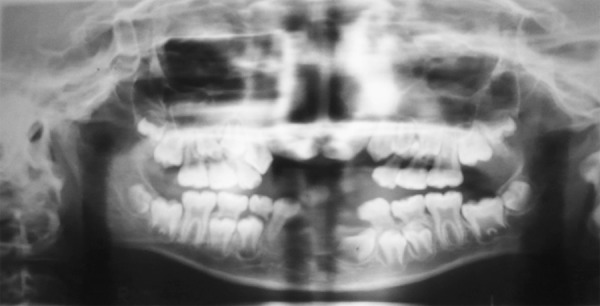
Radiograph showing absence of incisors

Middle portion of upper lip was deficient. Intraoral examination revealed fusion of middle portion of upper lip and gingiva (labiogingival attachment) (Figs 3 and 4). 11, 12, 21 and 22 were clinically missing with history of unerupted teeth ever, in maxillary and mandibular anterior region, which was confirmed with orthopantogram (Fig. 5).

**Fig. 6:**
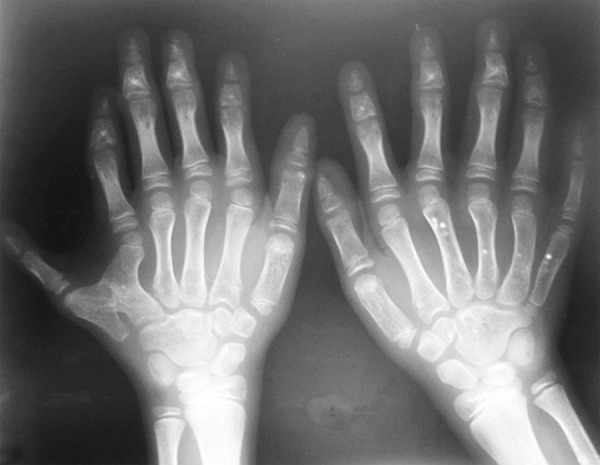
Radiograph showing postaxial polydactyly

**Fig. 7:**
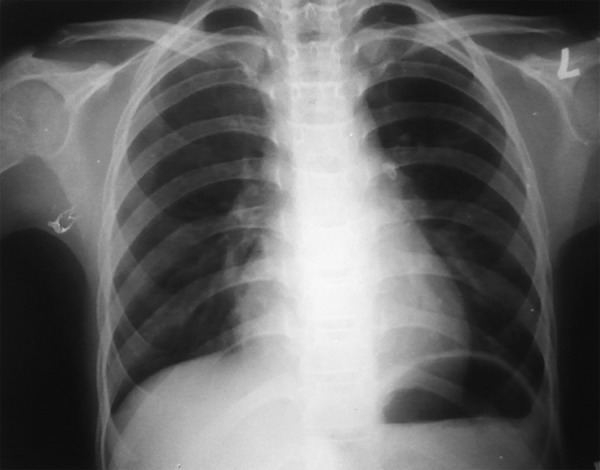
Radiograph showing cardiomegaly

Skeletal radiology of upper limb revealed short metacarpals, ulnar thick 5th metacarpal, ulnar polydactyly (Fig. 6). Chest X-ray showed cardiomegaly (Fig. 7).

Echocardiogram and electrocardiogram were performed which revealed atrial septal defect. Based on the history, clinical examination and investigations, a diagnosis of Ellis-van Creveld syndrome was made.

## DISCUSSION

EVCS is an autosomal recessive skeletal dysplasia. Although it is very common in Amish group, it does not show racial and gender predilection.^5^ The risk of recurrence for siblings is one in four (25%) for each offspring.^10^

Histopathologic examination of fetuses with EVCS revealed that the cartilage of long bones shows chondrocyte disorganization in the physeal growth zone. Variable chondrocyte disorganization was seen in the central physeal growth zone of the vertebrae.^6^ EVCS is one of the rare syndromes with a birth prevalence of 7/10, 00,000.^4^ The life expectancy is mainly determined by the congenital heart defect and the respiratory problems due to thoracic cage deformity. Patients who survive infancy have a normal life expectancy, the oldest living patient was 82 years of age.^12^

There is no sex predilection, in our case it is a male. There is also short limbed disproportionate dwarfism with the extremities shortened out of proportion to the trunk.^10^ In this case findings like disproportionate extremities, short stature and polydactyly were identified at the time of birth. The hands were short and wide exhibiting polydactyly with additional finger next to fifth finger, which is found in 100% of cases.

There is parental consanguinity in approximately 30% of the cases.^9^ For this patient parental consanguinity was positive but there was no history of similar sibling or family member. Neonatal history may include small size at birth, slow growth and skeletal anomalies as the initial symptoms. Natal teeth may be present.^6^ In our case there was a positive neonatal history of low birth weight, slow growth and skeletal anomalies but natal teeth were not present. Congenital heart defects occur in about 50% of cases. The most common is atrial septal defect and others are VSD and hypoplasia of aorta.^10^ In this case, atrial septal defect was confirmed. Although, most of the patients with EVCS have normal intelligence, mental retardation and central nervous system abnormalities were reported in some cases.^13^ Our patient had normal IQ level.

The nails are markedly hypoplastic, dystrophic, friable, thin and spoon-shaped and sometimes completely absent. The hair, particularly the eyebrows and the pubic hair, have been stated to be thin and sparse.^8^ Nails were hypoplastic, dystrophic in our patient.

Oral manifestations include fusion of middle portion of upper lip to the maxillary gingival margin eliminating the normal mucolateral sulcus. Intraorally, presence of natal and neonatal teeth and congenital absence of teeth particularly in mandibular anterior segment can be seen. Tooth eruption is delayed and those erupted are generally malformed or are affected by caries.^7^ In the present case, partial anodontia was present and enamel was normal in erupted teeth. Oral examination of this patient revealed absence of mucobuccal fold due to labiogingival attachment and deficient upper lip. All the positive findings of the patient confirmed the diagnosis of EVCS.

## CONCLUSION

Ellis-van Creveld syndrome is a rare autosomal recessive disorder with complex phenotype (disproportionate dwarfism and cardiac defects that might be life-threatening). Syndrome shows high mortality in early life, 1/3 of these patients die in infancy from cardiac and respiratory problems and those who survive require multidisciplinary approach for treatment. Early diagnosis and treatment can prevent the patient from various complications and undue psychological trauma.
